# Supplementary material for: Metabolic derangement in polycystic kidney disease mouse models is ameliorated by mitochondrial-targeted antioxidants
Source: Commun Biol. 2021 Oct 20;4:1200. doi: 10.1038/s42003-021-02730-w (PMC8528863; doi:10.1038/s42003-021-02730-w)
Supplement: Supplementary file 3 — Description of Additional Supplementary Files [file 42003_2021_2730_MOESM3_ESM.pdf]

Description of Additional Supplementary Files

File name: Supplementary Data 1

Description: Targeted proteomics profiling of mitochondrial and metabolic proteins from WT, RC/RC, and RC/null mice and RC/RC and RC/null mice treated with mCAT. Protein abundance and the magnitude of effect due to the PKD1 gene mutation, mCAT treatment, and PKD1 dose. Only the numbers with significant changes are shown. Positive denotes increases in abundance (in %) and negative denotes decreases in abundance (in %).

File name: Supplementary Data 2

Description: Source data for Figures 1-9.
