# Supplementary material for: Metabolic derangement in polycystic kidney disease mouse models is ameliorated by mitochondrial-targeted antioxidants
Source: Commun Biol. 2021 Oct 20;4:1200. doi: 10.1038/s42003-021-02730-w (PMC8528863; doi:10.1038/s42003-021-02730-w)
Supplement: Supplementary file 4 — Supplementary Data 1 [file 42003_2021_2730_MOESM4_ESM.pdf]

Protein ID	Protein name	Targeted Proteome Measurement of Abundance (pmol/100µg total protein)										Magnitude of Effect (%)*			
		WT		RC/RC		RC/RC+mCAT		RC/null		RC/null+mCAT		PKD1 mutation effect	mCAT effect	PKD1 dose effect	PKD1 dose & mCAT interaction
		Mean	SE	Mean	SE	Mean	SE	Mean	SE	Mean	SE				
**Fatty Acid Beta Oxidation (FAO)**															
**Fabp3**	fatty acid binding protein 3	0.384	0.028	0.234	0.016	0.441	0.124	0.160	0.041	0.157	0.007	-0.398	0.630	-0.416	-0.281
**Fabp4**	fatty acid binding protein 4	4.402	1.165	2.848	0.723	2.431	0.529	1.793	0.820	1.473	0.527	-0.448	-0.027	-0.381	-0.092
**Cpt1a**	carnitine palmitoyltransferase 1a	1.043	0.091	1.115	0.124	1.322	0.125	0.407	0.026	0.578	0.050	N.S	0.227	-0.613	0.154
**Cpt1b**	carnitine palmitoyltransferase 1b	0.170	0.019	0.092	0.010	0.140	0.019	0.028	0.008	0.058	0.020	-0.470	0.520	-0.727	0.386
**Cpt2**	carnitine palmitoyltransferase 2	0.268	0.029	0.200	0.025	0.208	0.022	0.072	0.016	0.097	0.006	-0.299	0.091	-0.637	0.322
**Hadh**	hydroxyacyl-Coenzyme A dehydrogenase(trifunctional protein)	2.987	0.445	2.758	0.460	2.990	0.366	0.681	0.086	0.776	0.003	-0.180	0.189	-0.723	N.S
**Hadha**	hydroxyacyl-CoA dehydrogenase/3-ketoacyl-CoA thiolase/enoyl-CoA hydratase	1.747	0.594	0.632	0.042	0.802	0.048	0.349	0.034	0.485	0.038	-0.595	0.316	-0.423	N.S
**Hadhb**	(trifunctional protein), alpha and beta subunit	1.262	0.319	0.535	0.032	0.604	0.051	0.299	0.049	0.363	0.030	-0.544	0.125	-0.452	0.110
**acsl1**	acyl-CoA synthetase long-chain family member 1	2.073	0.302	0.857	0.064	1.146	0.126	0.435	0.043	0.595	0.093	-0.583	0.333	-0.484	N.S
**Acad11**	acyl-Coenzyme A dehydrogenase family, member 11	0.413	0.088	0.391	0.051	0.475	0.049	0.092	0.023	0.129	0.041	N.S	0.262	-0.770	0.104
**Acadvl**	acyl-Coenzyme A dehydrogenase, very long chain	0.973	0.263	0.505	0.050	0.645	0.029	0.220	0.016	0.326	0.081	-0.454	0.354	-0.534	N.S
**Acadl**	acyl-Coenzyme A dehydrogenase, long-chain	1.533	0.394	1.091	0.096	1.408	0.059	0.515	0.034	0.712	0.172	-0.245	0.327	-0.516	N.S
**Acadm**	acyl-Coenzyme A dehydrogenase, medium chain	3.655	0.731	2.641	0.348	3.010	0.155	0.533	0.062	0.617	0.073	-0.298	0.216	-0.789	N.S
**Acads**	acyl-Coenzyme A dehydrogenase, short chain	0.469	0.068	0.633	0.089	0.662	0.049	0.172	0.011	0.173	0.011	0.194	0.098	-0.716	N.S
**Ehhadh**	enoyl-Coenzyme A, hydratase/3-hydroxyacyl Coenzyme A dehydrogenase	2.089	0.640	1.563	0.203	2.073	0.271	0.197	0.025	0.349	0.115	-0.168	0.388	-0.866	0.191
**echs1**	enoyl Coenzyme A hydratase, short chain, 1, mitochondrial	0.580	0.125	0.490	0.057	0.562	0.060	0.173	0.033	0.194	0.019	N.S	0.129	-0.656	N.S
**Eci1**	enoyl-Coenzyme A delta isomerase 1	0.561	0.094	0.469	0.057	0.532	0.049	0.205	0.028	0.218	0.016	-0.090	0.159	-0.562	N.S
**Eci2**	enoyl-Coenzyme A delta isomerase 2	0.075	0.015	0.059	0.006	0.064	0.003	0.036	0.004	0.044	0.008	-0.199	0.138	-0.358	N.S
**Acaa1a/b**	acetyl-Coenzyme A acyltransferase 1A/1B	0.140	0.024	0.101	0.012	0.130	0.011	0.062	0.011	0.075	0.008	-0.282	0.313	-0.400	N.S
**Acaa2**	acetyl-Coenzyme A acyltransferase 2 (mitochondrial 3-oxoacyl-CoA thiolase)	2.488	0.426	0.983	0.075	1.284	0.091	0.537	0.029	0.735	0.080	-0.601	0.335	-0.434	N.S
**crat**	carnitine acetyltransferase	0.198	0.058	0.109	0.011	0.126	0.008	0.044	0.005	0.056	0.005	-0.408	0.189	-0.587	0.085
**gpi**	glucose phosphate isomerase 1	0.243	0.020	0.166	0.024	0.143	0.022	0.170	0.030	0.240	0.003	-0.364	-0.120	0.064	0.678
**FA biosynthesis and ketogenesis**															
**decr1**	2,4-dienoyl CoA reductase 1, mitochondrial	1.313	0.177	1.160	0.136	1.247	0.133	0.425	0.057	0.513	0.037	N.S	0.052	-0.639	0.175
**Acot13**	acyl-CoA thioesterase 13	1.595	0.133	1.111	0.117	1.735	0.253	0.471	0.009	0.565	0.057	-0.326	0.535	-0.558	-0.226
**Hsd17b4**	hydroxysteroid (17-beta) dehydrogenase 4	1.001	0.195	1.006	0.131	1.047	0.118	0.440	0.055	0.468	0.040	N.S	0.079	-0.540	N.S
**Hmgcs1**	3-hydroxy-3-methylglutaryl-Coenzyme A synthase 1	0.071	0.007	0.105	0.013	0.119	0.015	0.072	0.008	0.070	0.002	0.394	0.156	-0.269	-0.152
**Hmgcs2**	3-hydroxy-3-methylglutaryl-Coenzyme A synthase 2	0.370	0.117	0.149	0.017	0.235	0.032	0.240	0.032	0.224	0.033	-0.553	0.562	0.646	-0.399
**HmgcL**	3-hydroxy-3-methylglutaryl-Coenzyme A lyase	0.879	0.147	0.866	0.119	1.019	0.123	0.306	0.026	0.302	0.028	N.S	0.209	-0.624	-0.183
**Bdh1**	3-hydroxybutyrate dehydrogenase, type 1	1.791	0.437	2.062	0.212	2.436	0.286	0.297	0.037	0.351	0.019	0.223	0.191	-0.852	N.S
**Glycolysis and Gluconeogenesis**															
**pygm**	muscle glycogen phosphorylase	0.025	0.003	0.014	0.002	0.031	0.011	0.010	0.004	0.033	0.010	-0.498	1.039	-0.291	0.670
**pygb**	brain glycogen phosphorylase	0.072	0.010	0.053	0.004	0.052	0.003	0.063	0.004	0.064	0.009	-0.259	N.S	0.214	N.S
**slc2a4**	solute carrier family 2 (facilitated glucose transporter), member 4	0.136	0.024	0.048	0.004	0.078	0.006	0.036	0.001	0.044	0.003	-0.639	0.619	-0.233	-0.254
**hk1**	hexokinase 1	0.531	0.097	0.477	0.077	0.405	0.021	0.508	0.089	0.406	0.054	-0.109	-0.113	N.S	N.S
**tkt**	transketolase	1.681	0.246	1.188	0.055	1.090	0.060	1.496	0.146	1.998	0.118	-0.273	-0.083	0.252	0.473
**Taldo1**	transaldolase 1	0.025	0.004	0.021	0.002	0.018	0.001	0.022	0.005	0.019	0.001	-0.163	-0.103	N.S	N.S
**gpi**	glucose phosphate isomerase 1	0.247	0.013	0.156	0.023	0.147	0.020	0.150	0.025	0.223	0.018	-0.419	N.S	N.S	0.552
**pfkl**	phosphofructokinase, liver, B-type	0.191	0.028	0.163	0.014	0.179	0.018	0.219	0.022	0.162	0.011	-0.106	N.S	0.310	-0.252
**pfkm**	phosphofructokinase, muscle	0.085	0.003	0.065	0.005	0.093	0.008	0.050	0.007	0.053	0.006	-0.251	0.429	-0.236	-0.255
**aldoa**	aldolase A, fructose-bisphosphate	1.439	0.328	0.995	0.051	1.004	0.054	1.198	0.162	1.076	0.054	-0.265	N.S	0.137	N.S
**aldob**	aldolase B, fructose-bisphosphate	9.498	1.824	8.474	1.146	9.561	0.539	3.192	0.457	3.286	0.208	-0.138	0.228	-0.599	-0.138
**tpi**	triosephosphate isomerase 1	0.744	0.091	0.781	0.095	0.768	0.081	0.572	0.056	0.546	0.033	N.S	N.S	-0.248	N.S
**gapdh**	glyceraldehyde-3-phosphate dehydrogenase	6.865	0.575	4.023	0.239	3.303	0.126	5.828	0.460	4.676	0.674	-0.412	-0.183	0.427	N.S
**pgk1**	phosphoglycerate kinase 1	1.152	0.126	0.848	0.063	0.849	0.027	0.563	0.054	0.395	0.031	-0.260	N.S	-0.337	-0.293
**pgam2**	phosphoglycerate mutase 2	0.417	0.141	0.239	0.035	0.309	0.035	0.097	0.010	0.141	0.022	-0.405	0.406	-0.546	N.S
**eno1**	enolase 1, alpha non-neuron	3.997	0.489	2.583	0.202	2.241	0.128	2.050	0.208	2.096	0.214	-0.368	-0.079	-0.145	N.S
**pkm2**	pyruvate kinase, muscle	2.313	0.247	1.311	0.100	1.353	0.044	1.939	0.216	1.798	0.145	-0.424	N.S	0.417	N.S
**ldha**	lactate dehydrogenase A	5.600	0.505	6.022	0.618	6.073	0.835	4.897	0.403	4.050	0.108	N.S	N.S	-0.149	-0.165
**ldhb**	lactate dehydrogenase B	5.692	0.701	5.343	0.506	6.018	0.572	2.982	0.432	2.687	0.096	N.S	0.113	-0.453	-0.162
**pc**	Pyruvate carboxylase	3.225	0.479	2.817	0.428	3.265	0.409	0.695	0.153	1.163	0.245	-0.213	0.248	-0.738	0.384
**mdh1**	malate dehydrogenase 1, NAD (soluble)	8.092	0.699	6.472	0.872	7.572	0.750	2.793	0.145	3.608	0.635	-0.253	0.233	-0.531	N.S
**hspd1**	heat shock protein 1 (chaperonin)	1.486	0.190	1.236	0.110	1.041	0.096	0.685	0.098	0.863	0.048	-0.178	-0.148	-0.443	0.523
**Antioxidants**															
**akr1b1**	aldo-keto reductase family 1, member B3 (aldose reductase)	1.357	0.222	0.410	0.100	0.552	0.097	0.290	0.013	0.273	0.007	-0.728	0.423	-0.174	-0.339
**aldh2**	aldehyde dehydrogenase 2, mitochondrial	2.440	0.284	2.396	0.208	2.619	0.147	1.051	0.106	1.657	0.285	N.S	0.108	-0.557	0.397
**cat**	Catalase	2.214	0.306	1.695	0.268	2.443	0.233	0.349	0.068	0.548	0.064	-0.318	0.638	-0.777	N.S
**sod1**	superoxide dismutase 1, soluble	1.193	0.212	0.942	0.126	1.275	0.151	0.419	0.040	0.505	0.121	N.S	0.266	-0.583	N.S
**sod2**	superoxide dismutase 2, mitochondrial	0.382	0.060	0.309	0.046	0.398	0.029	0.097	0.015	0.136	0.036	-0.231	0.378	-0.674	N.S
**gpi**	glucose phosphate isomerase 1	0.246	0.012	0.157	0.023	0.148	0.020	0.151	0.024	0.214	0.016	-0.416	N.S	N.S	0.488
**gpx1**	glutathione peroxidase 1	0.395	0.069	0.280	0.048	0.244	0.022	0.135	0.022	0.144	0.011	-0.345	N.S	-0.450	N.S
**gpx4**	glutathione peroxidase 4	0.370	0.042	0.398	0.040	0.520	0.055	0.190	0.011	0.209	0.052	N.S	0.249	-0.545	N.S
**gsr**	glutathione reductase	0.369	0.048	0.223	0.019	0.225	0.014	0.235	0.024	0.239	0.008	-0.379	N.S	N.S	N.S
**gsta3**	glutathione S-transferase, alpha 3	1.131	0.177	0.568	0.048	0.726	0.146	0.571	0.071	0.710	0.145	-0.494	0.202	N.S	N.S
**gstm1**	glutathione S-transferase, mu 1	4.889	0.921	3.992	0.426	4.238	0.456	3.261	0.458	3.065	0.564	N.S	N.S	-0.219	-0.063
**gstp1**	glutathione S-transferase, pi 1	0.495	0.088	0.624	0.085	0.665	0.084	0.431	0.075	0.368	0.040	N.S	N.S	-0.276	-0.123
**hspd1**	heat shock protein 1 (chaperonin)	1.486	0.190	1.236	0.110	1.041	0.096	0.685	0.098	0.837	0.022	N.S	N.S	-0.374	N.S
**mdh1**	malate dehydrogenase 1, NAD (soluble)	8.342	0.578	6.472	0.872	7.572	0.750	2.793	0.145	3.590	0.617	-0.278	0.232	-0.531	N.S
**msra**	methionine sulfoxide reductase A	1.005	0.151	0.864	0.111	0.994	0.123	0.297	0.062	0.265	0.007	N.S	0.076	-0.695	N.S
**phb**	prohibitin	0.658	0.053	0.671	0.092	0.506	0.090	0.424	0.077	0.342	0.022	N.S	-0.285	-0.325	N.S
**phb2**	prohibitin 2	0.571	0.100	0.470	0.083	0.272	0.038	0.274	0.042	0.218	0.015	-0.253	-0.334	-0.290	N.S
**prdx1**	peroxiredoxin 1	2.907	0.249	3.674	0.439	3.747	0.483	2.371	0.265	2.170	0.170	N.S	N.S	-0.311	-0.071
**prdx2**	peroxiredoxin 2	0.659	0.050	0.669	0.043	0.722	0.074	0.594	0.027	0.558	0.032	N.S	N.S	N.S	N.S
**prdx3**	peroxiredoxin 3	0.732	0.087	0.638	0.083	0.675	0.069	0.265	0.036	0.259	0.018	N.S	N.S	-0.600	N.S
**prdx5**	peroxiredoxin 5	4.485	1.005	3.569	0.684	4.191	0.610	1.219	0.049	0.891	0.052	-0.267	0.289	-0.606	-0.433
**prdx6**	peroxiredoxin 6	0.321	0.045	0.272	0.033	0.252	0.025	0.205	0.029	0.218	0.013	-0.192	N.S	-0.178	N.S
**txn1**	thioredoxin 1	1.603	0.138	1.653	0.169	2.111	0.302	2.046	0.215	1.429	0.062	N.S	0.184	N.S	-0.282
**txnrd1**	thioredoxin reductase 1	0.256	0.021	0.226	0.014	0.255	0.017	0.216	0.011	0.195	0.021	N.S	N.S	-0.097	-0.103
**TCA cycle and Related Proteins**															
**pdha1**	pyruvate dehydrogenase E1 alpha 1	1.630	0.345	1.099	0.112	1.171	0.070	0.423	0.049	0.457	0.005	-0.314	0.105	-0.604	N.S
**pdhb**	pyruvate dehydrogenase (lipoamide) beta	1.199	0.260	0.571	0.048	0.707	0.057	0.297	0.038	0.342	0.035	-0.506	0.257	-0.473	-0.074
**pdk1**	pyruvate dehydrogenase kinase, isoenzyme 1	0.019	0.004	0.012	0.001	0.011	0.001	0.009	0.001	0.011	0.001	-0.349	-0.066	-0.291	0.364
**pdk2**	pyruvate dehydrogenase kinase, isoenzyme2	0.073	0.011	0.048	0.004	0.044	0.004	0.022	0.003	0.023	0.003	-0.348	-0.048	-0.530	0.112
**dlat**	dihydrolipoamide S-acetyltransferase (E2 component of PDH)	1.879	0.397	1.100	0.122	1.288	0.091	0.597	0.038	0.746	0.055	-0.414	0.227	-0.424	N.S
**dld**	dihydrolipoamide dehydrogenase	0.769	0.082	0.462	0.052	0.573	0.051	0.195	0.032	0.263	0.016	-0.422	0.280	-0.572	0.094
**dlst**	dihydrolipoamide S-succinyltransferase (E2 comp of 2-oxo-glutarate complex)	2.608	0.380	2.226	0.301	2.328	0.276	0.726	0.099	0.814	0.044	-0.194	0.099	-0.654	0.048
**cs**	citrate synthase	3.017	0.862	1.426	0.096	1.700	0.155	0.688	0.030	0.976	0.116	-0.480	0.188	-0.509	0.180
**aco2**	aconitase 2, mitochondrial	4.360	0.896	2.466	0.325	2.812	0.219	0.627	0.113	0.890	0.073	-0.442	0.211	-0.739	0.226
**idh1**	isocitrate dehydrogenase 1 (NADP+), soluble	3.783	0.316	2.429	0.187	2.847	0.143	1.626	0.093	1.379	0.080	-0.369	0.195	-0.316	-0.289
**idh2**	isocitrate dehydrogenase 2 (NADP+), mitochondrial	5.181	1.048	3.146	0.264	3.822	0.258	1.201	0.138	1.497	0.188	-0.377	0.242	-0.611	N.S
**idh3a**	isocitrate dehydrogenase 3 (NAD+) alpha	0.560	0.085	0.393	0.035	0.395	0.020	0.178	0.006	0.228	0.011	-0.299	0.036	-0.531	0.240
**idh3b**	isocitrate dehydrogenase 3 (NAD+) beta	0.472	0.068	0.309	0.018	0.344	0.021	0.212	0.011	0.268	0.016	-0.337	0.118	-0.306	0.132
**idh3g**	isocitrate dehydrogenase 3 (NAD+), gamma	1.100	0.189	0.748	0.072	0.826	0.036	0.325	0.018	0.397	0.043	-0.317	0.146	-0.549	0.059
**ogdh**	oxoglutarate (alpha-ketoglutarate) dehydrogenase (lipoamide)	1.599	0.266	1.093	0.121	1.259	0.110	0.403	0.029	0.568	0.017	-0.324	0.187	-0.615	0.198
**suclg1**	succinate-CoA ligase, GDP-forming, alpha subunit	0.607	0.105	0.534	0.071	0.585	0.051	0.178	0.023	0.229	0.021	-0.163	0.170	-0.644	0.116
**sucla2**	succinate-Coenzyme A ligase, ADP-forming, beta subunit	1.067	0.144	0.717	0.060	0.780	0.037	0.321	0.023	0.365	0.042	-0.332	0.121	-0.539	N.S
**sdha**	succinate dehydrogenase complex, subunit A, flavoprotein (Fp)	2.519	0.254	2.030	0.248	2.567	0.129	0.635	0.034	0.768	0.065	-0.235	0.343	-0.667	-0.102
**sdhb**	succinate dehydrogenase complex, subunit B, iron sulfur (Ip)	0.568	0.063	0.453	0.057	0.461	0.045	0.157	0.020	0.234	0.010	-0.242	0.061	-0.638	0.436
**sdhc**	succinate dehydrogenase complex, subunit C, integral membrane protein	0.220	0.019	0.201	0.026	0.265	0.033	0.100	0.002	0.099	0.006	-0.138	0.350	-0.466	-0.274
**fh1**	fumarate hydratase 1	0.741	0.121	0.585	0.060	0.629	0.028	0.225	0.027	0.249	0.022	-0.217	0.120	-0.605	N.S
**mdh1**	malate dehydrogenase 1, NAD (soluble)	7.830	0.500	6.555	0.800	7.529	0.744	2.882	0.152	3.514	0.643	-0.212	0.190	-0.533	N.S

**mdh2**	malate dehydrogenase 2, NAD (mitochondrial)	2.671	0.513	1.775	0.265	1.716	0.173	0.670	0.074	0.833	0.026	-0.363	0.030	-0.590	0.228
**Glud1**	glutamate dehydrogenase 1	2.362	0.897	1.534	0.152	1.850	0.115	0.917	0.186	0.950	0.019	N.S	0.214	-0.426	-0.095
**got1**	glutamic-oxaloacetic transaminase 1, soluble	0.664	0.223	0.310	0.022	0.342	0.037	0.170	0.023	0.215	0.029	-0.475	0.090	-0.454	0.164
**got2**	glutamatic-oxaloacetic transaminase 2, mitochondrial	1.697	0.286	1.190	0.104	1.217	0.099	0.563	0.089	0.737	0.021	-0.291	0.037	-0.526	0.301
**Respiratory Complexes and Related Proteins**															
**Ndufs1**	NADH dehydrogenase (ubiquinone) Fe-S protein 1	1.107	0.276	0.800	0.069	0.628	0.097	0.338	0.018	0.425	0.023	-0.240	-0.271	-0.566	0.725
**Ndufv1**	NADH dehydrogenase (ubiquinone) flavoprotein 1	0.888	0.194	0.690	0.052	0.585	0.091	0.325	0.024	0.341	0.032	-0.188	-0.209	-0.522	0.325
**sdha**	succinate dehydrogenase complex, subunit A, flavoprotein (Fp)	2.519	0.254	2.030	0.248	2.567	0.129	0.635	0.034	0.768	0.065	-0.235	0.343	-0.667	-0.102
**sdhb**	succinate dehydrogenase complex, subunit B, iron sulfur (Ip)	0.568	0.063	0.453	0.057	0.461	0.045	0.157	0.020	0.234	0.010	-0.242	0.061	-0.638	0.436
**sdhc**	succinate dehydrogenase complex, subunit C, integral membrane protein	0.220	0.019	0.201	0.026	0.265	0.033	0.100	0.002	0.099	0.006	-0.138	0.350	-0.466	-0.274
**etfa**	electron transferring flavoprotein, alpha polypeptide	1.260	0.276	0.797	0.115	1.110	0.112	0.243	0.024	0.387	0.054	-0.377	0.480	-0.672	0.069
**etfb**	electron transferring flavoprotein, beta polypeptide	2.549	0.572	1.863	0.225	2.177	0.230	0.628	0.086	0.708	0.076	-0.262	0.217	-0.647	-0.060
**etfdh**	electron transferring flavoprotein, dehydrogenase	0.835	0.197	0.531	0.064	0.645	0.057	0.216	0.018	0.251	0.010	-0.360	0.271	-0.568	-0.079
**Coq6**	coenzyme Q6 homolog (yeast)	0.040	0.009	0.025	0.003	0.031	0.003	0.017	0.002	0.015	0.001	-0.364	0.283	-0.302	-0.299
**Uqcrc1**	ubiquinol-cytochrome c reductase core protein 1	2.984	0.619	2.244	0.206	2.157	0.201	0.625	0.062	0.865	0.131	-0.234	-0.035	-0.715	0.423
**Atp5a1**	ATP synthase, H+ transporting, mitochondrial F1 complex, alpha subunit 1	14.823	1.367	9.017	1.192	10.672	0.923	5.417	0.734	6.904	0.634	-0.427	0.243	-0.374	0.046
**Atp5b**	ATP synthase, H+ transporting mitochondrial F1 complex, beta subunit	16.525	1.622	11.896	1.169	12.496	0.960	4.973	0.705	6.771	0.593	-0.298	0.072	-0.578	0.298
**Proteostasis Pathways**															
**Cryab**	crystallin, alpha B	0.142	0.020	0.044	0.007	0.064	0.003	0.044	0.010	0.056	0.012	-0.707	0.578	N.S	-0.171
**hsp90b1**	heat shock protein 90, beta (Grp94), member 1	1.056	0.142	1.293	0.097	1.112	0.090	1.236	0.251	1.243	0.120	0.157	N.S	N.S	N.S
**hspa1a**	heat shock protein 1A	1.974	0.110	1.568	0.137	1.491	0.119	1.451	0.224	1.497	0.295	N.S	N.S	-0.106	N.S
**hspa5**	heat shock protein 5	0.291	0.016	0.270	0.030	0.192	0.021	0.253	0.060	0.291	0.034	-0.139	-0.253	N.S	0.542
**hspa9**	heat shock protein 9	0.785	0.163	0.611	0.102	0.561	0.069	0.217	0.045	0.235	0.020	-0.160	N.S	-0.605	N.S
**hspd1**	heat shock protein 1 (chaperonin)	1.407	0.194	1.259	0.108	1.023	0.102	0.687	0.115	0.792	0.016	N.S	-0.214	-0.479	0.532
**lonp1**	lon peptidase 1, mitochondrial	0.205	0.046	0.176	0.015	0.204	0.011	0.095	0.006	0.117	0.007	N.S	0.168	-0.449	N.S
**lonp2**	lon peptidase 2, peroxisomal	0.023	0.003	0.021	0.004	0.024	0.002	0.008	0.003	0.012	0.004	-0.205	0.332	-0.625	0.175
**Peroxisomal Proteins**															
**Abcd3**	ATP-binding cassette, sub-family D (ALD), member 3	0.465	0.058	0.544	0.071	0.645	0.090	0.217	0.022	0.200	0.015	N.S	0.262	-0.560	-0.262
**Acox1**	acyl-Coenzyme A oxidase 1, palmitoyl	0.327	0.063	0.429	0.071	0.487	0.075	0.087	0.008	0.142	0.040	N.S	0.278	-0.752	0.187
**Ech1**	enoyl coenzyme A hydratase 1, peroxisomal	0.927	0.127	0.917	0.118	1.104	0.101	0.366	0.031	0.392	0.016	N.S	0.247	-0.580	-0.134
**Ephx2**	Epoxide Hydrolase 2	0.783	0.141	1.790	0.357	2.004	0.342	0.317	0.110	0.326	0.016	0.820	0.289	-0.809	N.S
**mdh1**	malate dehydrogenase 1, NAD (soluble)	8.040	0.796	6.655	0.859	7.770	0.741	2.961	0.234	3.708	0.597	-0.218	0.221	-0.525	N.S
**Pecr**	peroxisomal trans-2-enoyl-CoA reductase	2.617	0.911	3.403	0.702	3.605	0.611	0.254	0.086	0.394	0.128	0.269	0.276	-0.918	0.333
**slc25a20**	solute carrier family 25 (mitochondrial carnitine/acylcarnitine translocase) 20	0.485	0.090	0.330	0.027	0.274	0.025	0.195	0.018	0.220	0.020	-0.305	-0.170	-0.400	0.363
**Other Mitochondrial Proteins**															
**Atp2a2**	ATPase, Ca++ transporting, cardiac muscle, slow twitch 2	0.139	0.010	0.138	0.009	0.159	0.035	0.117	0.003	0.179	0.018	N.S	0.046	-0.139	0.443
**cd36**	CD36 antigen	0.151	0.019	0.159	0.026	0.136	0.024	0.034	0.005	0.041	0.007	-0.071	-0.106	-0.761	0.345
**Clpp**	caseinolytic mitochondrial matrix peptidase proteolytic subunit	0.214	0.047	0.266	0.029	0.283	0.036	0.121	0.020	0.133	0.003	0.269	0.077	-0.537	0.059
**Clpx**	caseinolytic mitochondrial matrix peptidase chaperone subunit	0.105	0.013	0.121	0.011	0.122	0.008	0.127	0.005	0.146	0.007	0.137	0.035	0.089	0.109
**ckmt1**	creatine kinase, mitochondrial 2	1.898	1.389	0.473	0.079	0.568	0.091	0.314	0.148	0.245	0.068	-0.543	0.227	-0.460	-0.100
**prkaca**	protein kinase, cAMP dependent, catalytic, alpha	0.069	0.006	0.052	0.003	0.050	0.003	0.059	0.003	0.061	0.002	-0.239	-0.032	0.133	0.076
**Rhot1**	Ras Homolog Family Member T1	0.162	0.012	0.144	0.012	0.159	0.010	0.076	0.002	0.096	0.003	-0.129	0.127	-0.459	0.130
**Samm50**	sorting and assembly machinery component 50 homolog (S. cerevisiae)	0.395	0.039	0.275	0.016	0.248	0.036	0.167	0.018	0.209	0.011	-0.305	-0.149	-0.394	0.491
**slc25a11**	solute carrier family 25 (mitochondrial carrier oxoglutarate carrier), member 11	0.925	0.231	0.662	0.069	0.627	0.050	0.297	0.017	0.364	0.022	-0.266	N.S	-0.529	0.229
**Slc25a4**	solute carrier family 25 (mitochondrial carrier, adenine nucleotide translocator), member	1.932	0.029	1.348	0.091	1.914	0.302	0.909	0.038	1.201	0.074	-0.313	0.348	-0.324	N.S
**Slc25a4/5/31**	solute carrier family 25 (mitochondrial carrier, adenine nucleotide translocator), member	41.064	3.015	33.056	2.755	38.219	2.826	13.184	0.955	18.025	2.460	-0.211	0.171	-0.592	0.153
**Tufm**	Tu translation elongation factor, mitochondrial	0.412	0.079	0.357	0.031	0.360	0.024	0.132	0.010	0.192	0.019	-0.118	0.024	-0.622	0.416

*** The magnitude of effects are calculated only for those with significant changes; NS= not significant**
