# Supplementary material for: Metabolic derangement in polycystic kidney disease mouse models is ameliorated by mitochondrial-targeted antioxidants
Source: Commun Biol. 2021 Oct 20;4:1200. doi: 10.1038/s42003-021-02730-w (PMC8528863; doi:10.1038/s42003-021-02730-w)
Supplement: Supplementary file 6 — Reporting Summary [file 42003_2021_2730_MOESM6_ESM.pdf]

nature portfolio

Corresponding author(s): Dao-Fu Dai

Last updated by author(s): Aug 17, 2021

Reporting Summary

Nature Portfolio wishes to improve the reproducibility of the work that we publish. This form provides structure for consistency and transparency in reporting. For further information on Nature Portfolio policies, see our Editorial Policies and the Editorial Policy Checklist.

Statistics

For all statistical analyses, confirm that the following items are present in the figure legend, table legend, main text, or Methods section.

n/a	Confirmed	
☐	☒	The exact sample size (*n*) for each experimental group/condition, given as a discrete number and unit of measurement
☐	☒	A statement on whether measurements were taken from distinct samples or whether the same sample was measured repeatedly
☐	☒	The statistical test(s) used AND whether they are one- or two-sided<br>*Only common tests should be described solely by name; describe more complex techniques in the Methods section.*
☐	☒	A description of all covariates tested
☐	☒	A description of any assumptions or corrections, such as tests of normality and adjustment for multiple comparisons
☐	☒	A full description of the statistical parameters including central tendency (e.g. means) or other basic estimates (e.g. regression coefficient) AND variation (e.g. standard deviation) or associated estimates of uncertainty (e.g. confidence intervals)
☐	☒	For null hypothesis testing, the test statistic (e.g. *F*, *t*, *r*) with confidence intervals, effect sizes, degrees of freedom and *P* value noted<br>*Give P values as exact values whenever suitable.*
☒	☐	For Bayesian analysis, information on the choice of priors and Markov chain Monte Carlo settings
☒	☐	For hierarchical and complex designs, identification of the appropriate level for tests and full reporting of outcomes
☒	☐	Estimates of effect sizes (e.g. Cohen's *d*, Pearson's *r*), indicating how they were calculated

Our web collection on statistics for biologists contains articles on many of the points above.

Software and code

Policy information about availability of computer code

Data collection	No software was used
Data analysis	STATA, R, Graphpad Prism

For manuscripts utilizing custom algorithms or software that are central to the research but not yet described in published literature, software must be made available to editors and reviewers. We strongly encourage code deposition in a community repository (e.g. GitHub). See the Nature Portfolio guidelines for submitting code & software for further information.

Data

Policy information about availability of data

All manuscripts must include a data availability statement. This statement should provide the following information, where applicable:

- Accession codes, unique identifiers, or web links for publicly available datasets
- A description of any restrictions on data availability
- For clinical datasets or third party data, please ensure that the statement adheres to our policy

The source data underlying the targeted proteomics are provided in the Supplementary table 1 and the source data underlying the graphs are provided as a seperate source file. Other relevant data are available from the corresponding author upon request.

Field-specific reporting

Please select the one below that is the best fit for your research. If you are not sure, read the appropriate sections before making your selection.

- [x] Life sciences
- [ ] Behavioural & social sciences
- [ ] Ecological, evolutionary & environmental sciences

For a reference copy of the document with all sections, see nature.com/documents/nr-reporting-summary-flat.pdf

Life sciences study design

All studies must disclose on these points even when the disclosure is negative.

Sample size	Since the variance of several parameters tested in this project is unknown, we did not calculate the sample size. However, we try to use an arbitrary sample size of at least 5 for mouse experiments. Some experiments (e.g. targeted proteomics and Seahorse) are very expensive, so that there was a limit of samples that were done. In brief, many of the parameters show significant changes. We admit that some tests may be underpowered (inadequate) to significantly detect small changes among groups.
Data exclusions	No data were excluded from the analysis
Replication	All provided data are reproducible and all attempts at replication were successful.
Randomization	Samples (mice) were allocated into experimental groups randomly.
Blinding	Investigators were blinded to group allocation

Reporting for specific materials, systems and methods

We require information from authors about some types of materials, experimental systems and methods used in many studies. Here, indicate whether each material, system or method listed is relevant to your study. If you are not sure if a list item applies to your research, read the appropriate section before selecting a response.

Materials & experimental systems

n/a	Involved in the study	
	x	Antibodies
	x	Eukaryotic cell lines
x		Palaeontology and archaeology
	x	Animals and other organisms
x		Human research participants
x		Clinical data
x		Dual use research of concern

Methods

n/a	Involved in the study	
x		ChIP-seq
x		Flow cytometry
x		MRI-based neuroimaging

Antibodies

Antibodies used	AMPK (Cell Signaling Technology, 2532, polyclonal), phospho-AMPK (Thr172) (Cell Signaling Technology, 2531, polyclonal), p44/42 MAPK (Erk1/2) (Cell Signaling Technology, 4695, monoclonal), phospho-p44/42 MAPK (ERK1/2) (Cell Signaling Technology, 4370, monoclonal), Ki-67 (abcam, ab16667, monoclonal), anti-Catalase (R&D, AF3398, polyclonal), anti-rabbit IgG-HRP (Santa Cruz Biotechnology; sc-2357, monoclonal), anti-mouse IgG-HRP (Abcam; ab97046,polyclonal).
Validation	The AMPK antibody is validated to be used for western blot in both human and mouse species according to cell signaling technology website. The phospho-AMPK (Thr172) antibody is validated to be used for western blot in both human and mouse species according to cell signaling technology website. The p44/42 MAPK (Erk1/2) antibody is validated to be used for western blot in both human and mouse species according to cell signaling technology website. phospho-p44/42 MAPK (ERK1/2) antibody is validated to be used for western blot in both human and mouse species according to cell signaling technology website. The anti-rabbit IgG-HRP is validated to be used for western blot according to santa cruz biotechnology website. The anti-mouse IgG-HRP is validated to be used for western blot according to abcam website.

Eukaryotic cell lines

Policy information about cell lines

Cell line source(s)	WT 9-7 (ATCC® CRL-2830™), HK-2 (ATCC® CRL-2190™)
Authentication	Authentication and certificate of analysis were done by ATCC and the CoA are available upon request.

Mycoplasma contamination	This was performed by ATCC
Commonly misidentified lines (See ICLAC register)	*Name any commonly misidentified cell lines used in the study and provide a rationale for their use.*

Animals and other organisms

Policy information about studies involving animals; ARRIVE guidelines recommended for reporting animal research

Laboratory animals	Pkd1 p.R3277C knock-in mice (male and female, 6-8 months age) were obtained from Mayo clinic. The PKD1+/- mice generated by breeding PKD1flox/flox mice (B6.129S4-Pkd1tm2Ggg/J, JAX, 129S4/SvJae background) with germline Sox2-Cre transgenic mice (male and female, 4-8months old) (Edil3Tg(Sox2-cre)1Amc, JAX, C57BL/6 background); The PKD1RC/null and RC/WT mice were generated by crossing PKD1RC/RC mice with PKD1+/- mice (male and female, 20-23 days old)
Wild animals	Study did not involve wild animals
Field-collected samples	*For laboratory work with field-collected samples, describe all relevant parameters such as housing, maintenance, temperature, photoperiod and end-of-experiment protocol OR state that the study did not involve samples collected from the field.*
Ethics oversight	Animal experiments were approved by the University of Iowa Animal Care and Use Committee.

Note that full information on the approval of the study protocol must also be provided in the manuscript.
